# A systematic review of the measurement properties of aspects of psychological capacity in older adults

**DOI:** 10.1093/ageing/afad100

**Published:** 2023-10-30

**Authors:** Candice Oster, Sonia Hines, Chris Rissel, Dennis Asante, Jyoti Khadka, Katrin M Seeher, Jotheeswaran Amuthavalli Thiyagarajan, Christopher Mikton, Theresa Diaz, Vivian Isaac

**Affiliations:** Caring Futures Institute, College of Nursing & Health Sciences, Flinders University, Adelaide, Australia; College of Medicine & Public Health, Flinders Rural and Remote Health, Flinders University, Alice Springs, Northern Territory, Australia; College of Medicine & Public Health, Flinders Rural and Remote Health, Flinders University, Alice Springs, Northern Territory, Australia; College of Medicine & Public Health, Flinders Rural and Remote Health, Flinders University, Alice Springs, Northern Territory, Australia; Caring Futures Institute, College of Nursing & Health Sciences, Flinders University, Adelaide, Australia; Brain Health Unit, Department of Mental Health and Substance Use, World Health Organization, Geneva, Switzerland; Ageing and Health Unit, Department of Maternal, Newborn, Child, Adolescent Health and Ageing, World Health Organization, Geneva, Switzerland; Demographic Change and Healthy Ageing Unit, Social Determinants of Health, World Health Organization, Geneva, Switzerland; Epidemiology, Monitoring and Evaluation Unit, Department of Maternal, Newborn, Child, Adolescent Health and Ageing, World Health Organization, Geneva, Switzerland; College of Medicine & Public Health, Flinders Rural and Remote Health, Flinders University, Alice Springs, Northern Territory, Australia; School of Allied Health, Exercise & Sports Sciences, Faculty of Science & Health, Charles Sturt University, Albury, New South Wales, Australia

**Keywords:** healthy ageing, intrinsic capacity, psychological capacity, psychometric property, systematic review, older people

## Abstract

**Objective:**

to examine the measurement properties of instruments that have been used to measure aspects of psychological capacity in adults aged 60 years and over.

**Methods:**

the databases PsycINFO, MEDLINE, EMCARE and Scopus from 2010 were searched using search terms related to psychological capacity, older persons and measurement properties. Both data extraction and risk-of-bias assessment were conducted using the COSMIN (COnsensus-based Standards for the selection of health Measurement INstruments) criteria using Covidence software.

**Results:**

the full text of 326 articles were reviewed and a total of 30 studies were included, plus two further articles identified from reference lists (*n* = 32). No single instrument measuring psychological capacity was identified. Twenty (*n* = 20) instruments were identified that measure seven constructs of psychological capacity: Resilience; Sense of coherence; Hope; Mindfulness; Optimism; Attachment to life; Emotional regulation.

**Conclusions:**

this systematic review identified potential measures of psychological capacity in older adults. The review will inform further work to develop a single comprehensive measure of psychological capacity in older adults.

## Key Points

- No single measure encompassing the broad concept of intrinsic psychological capacity was identified in the review.
- The review identified 20 instruments measuring 7 constructs of psychological capacity.
- There is lack of definitional clarity of the concept of psychological capacity, requiring further work.

## Introduction

The United Nations Decade of Healthy Ageing (2021–30) calls for strengthening measurement for monitoring the healthy ageing and well-being of older people, including the measurement of psychological capacity [1]. In 2015, the World Health Organization (WHO) released a public health framework for healthy ageing [2]. Rather than considering healthy ageing from the perspective of the presence or absence of disease, WHO’s functioning-based approach is oriented around building and maintaining the ability of older people to be, and to do, the things they have reason to value. WHO proposes that this ‘functional ability’ is determined by the ‘intrinsic capacity’ of the individual, the environments in which they live, and the interaction between the individual and these environments. Intrinsic capacity is defined as ‘all the physical and mental capacities’ that an individual can draw on at any point in time. According to WHO’s measurement model, intrinsic capacity consists of five domains, namely cognitive, psychological, locomotor, sensory and vitality [3].

While there is some clarity on the structure of intrinsic capacity and its subdomains, clear conceptual and operational definitions of psychological capacity are still lacking. WHO states that psychological capacity is ‘mostly related to emotional functions’, particularly depression, but also includes aspects such as anxiety, personality characteristics, coping and mastery [2]. Recent validation studies considered measures of affect, sleep, depression, self-reported life satisfaction, locus of control and social participation as attributes of psychological capacity [3–6]. A growing body of evidence from positive psychology suggests a broader set of attributes such as self-efficacy, resilience, hope, optimism, sense of coherence, autonomy, resourcefulness, identity, hope, religiosity/spirituality and life valuation [7] are associated with older persons’ functional ability and well-being [8–10].

There is a better understanding of psychometric properties of measures of depression in older adults; however, the measurement properties of positive psychological measures of psychological capacity are poorly understood [11–13]. To our best knowledge, there are no systematic reviews that have assessed the measurement properties of instruments to measure different attributes of psychological capacity. This review will focus on positive psychological measures that could then be used to develop sub-dimensions of a comprehensive measure of psychological capacity. Therefore, we aim to examine the measurement properties of instruments used to measure different attributes of psychological capacity in older persons aged 60 years and over. The focus is on psychological capacity understood as positive states of mind, characterised by attributes such as resilience, hope, self-efficacy and optimism.

## Aim

The aim of the review was to examine the psychometric properties of instruments that have been used to measure aspects of psychological capacity in adults aged 60 years and over.

## Methods

### Search strategy

The databases PsycINFO (OVID interface, 1806 onwards), MEDLINE (OVID interface, 1946 onwards), EMCARE (OVID interface, 1995 onwards) and Scopus were searched using combinations of subject headings and free-text words. Search terms related to psychological capacity, older persons and measurement properties (see Appendix 1). Reference lists of included studies were also screened for potentially relevant articles. In addition, websites of two major institutions (Global Gateway for Ageing https://g2aging.org/ and Maelstrom https://www.maelstrom-research.org/network/near) that document the measures used in population surveys on ageing were searched to identify potential instruments and manuals. The review was restricted for year of publication from 2010 onwards. All database searches were conducted between July and October 2022. The study protocol is published in PROSPERO (CRD42022299578).

### Study selection

Inclusion criteria:

(i) Population: Older adults aged 60 years or over living in the community or long-term care facilities. Studies must include ≥50% aged 60 or older.
(ii) Instruments: Tools measuring aspects of psychological capacity as an individual’s inherent positive psychological state were eligible for inclusion.
(iii) Constructs: Constructs were eligible for inclusion if they measured aspects of positive psychological capacity as positive beliefs, thoughts and feelings that enable individuals to enhance their physical, mental and social functioning. In our review, psychological capacity does not include mood, depression and outcomes. Furthermore, psychological capacity does not include individuals’ outcome evaluation of their cognitive functions, health outcomes, satisfaction, quality of life or wellbeing. Potential constructs were reviewed, and consensus was reached through an iterative process in team meetings based on the following criteria: (a) positive psychological construct grounded in theory and research; (b) valid measurement; (c) dynamic positive state of mind; and (d) have a positive impact on health and wellbeing.
(iv) Outcomes: Psychometric properties such as internal consistency, reliability, measurement error, structural validity, hypothesis testing, cross-cultural validity, measurement invariance, criterion validity and responsiveness.
(v) Study types: Peer-reviewed studies that assess one or more psychometric properties of instruments for measuring psychological capacity in people aged 60 and over. Only studies published in English were included.

Exclusion criteria:

(i) Psychological capacity was measured as proxy (e.g. caregivers).
(ii) Psychological capacity measurement was a sub-scale, and the results of the psychometric properties were not reported separately.
(iii) Those instruments measuring psychological capacities for specific disease states or situations, such as coping in cancer or social contact self-efficacy, were not considered to be eligible for inclusion.
(iv) Unpublished articles, or articles for which the full text was not available.
(v) Reviews, qualitative studies or protocol articles.
(vi) Non-English language.

Search results were exported to Covidence systematic review software (Veritas Health Innovation, Melbourne, Australia) and duplicates removed. Titles and abstracts were screened for congruence to the inclusion criteria by two independent reviewers and differences of opinion adjudicated by a third reviewer where necessary. Full texts of the remaining citations were retrieved and assessed for inclusion by two independent reviewers.

### Data extraction and risk-of-bias assessment

Both data extraction and risk-of-bias assessment were conducted using the COSMIN (COnsensus-based Standards for the selection of health Measurement INstruments) criteria [14] using Covidence. Two reviewers independently extracted and assessed each paper and conflicts were adjudicated by a third reviewer.

### Data synthesis

Data were collated and described in tables and a narrative description developed. Findings were made on the quality (reliability, validity, consistency and responsiveness) of individual instruments and used to draw conclusions and make recommendations for future work. The validity and reliability of each included instrument were summarised using the updated criteria for good measurement properties described by Prinsen *et al*. [15].

## Results

### Study inclusion

**Figure 1:**
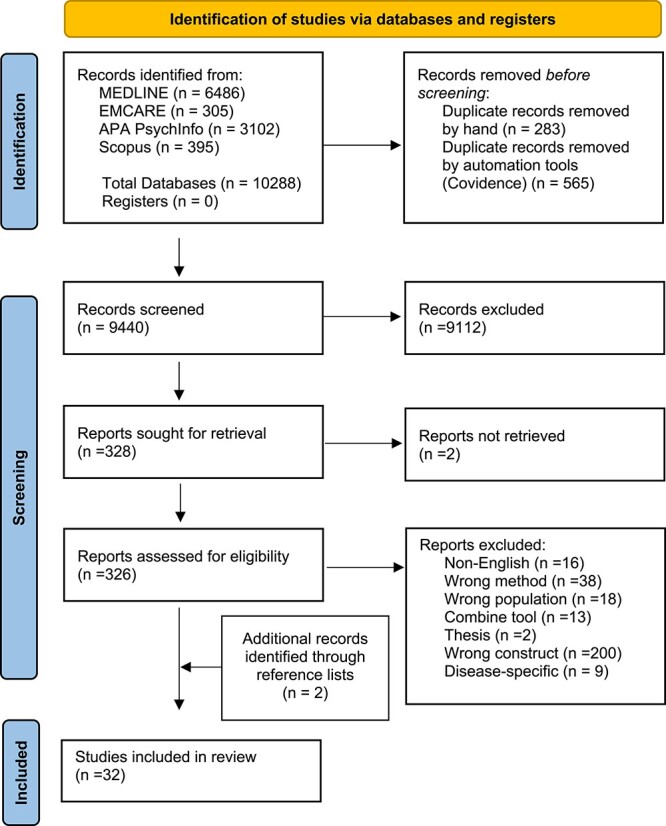
PRISMA flowchart.

The database searches identified 10,288 potentially relevant citations. Following removal of duplicates, the title and abstract of 9,440 citations were checked against the inclusion criteria. The full text of 326 articles were assessed for congruence with the review and a total of 30 studies were included. Two further articles were identified from the reference lists of relevant studies and included for a total of 32. Results of the search and study selection procedure are shown in (Figure 1).

No single instrument measuring psychological capacity was identified. Twenty (*n* = 20) instruments were identified that measure seven constructs of psychological capacity: Resilience; Sense of coherence; Hope; Mindfulness; Optimism; Attachment to Life; Emotional regulation.

### Characteristics of included studies

Characteristics of the included studies are presented in Table 1. Most studies (*n* = 31, 97%) were cross-sectional, with one cohort study included. The studies were conducted between 2011 and 2022. The number of participants in the studies ranged from 64 [16] to 4,604 [17]. The mean age in the studies ranged from 63 [18, 19] to 86 [20]. There was a disproportionate ratio of males to females in 23 (72%) studies with all but three of these having a larger proportion of female participants. Participants were mainly recruited from community settings (*n* = 26, 81%) followed by aged care facilities (*n* = 3), hospital (*n* = 2) and primary care (*n* = 1). Eight studies (25%) reported participant health conditions.

The studies were conducted across 17 countries with the most represented countries being the USA (*n* = 7), Spain (*n* = 4) and China (*n* = 4). The countries were mostly high income (*n* = 25, 78%) (according to the World Bank country classifications) followed by upper middle-income (*n* = 6, 19%). One study was conducted in a lower middle-income country (Iran) and no low-income countries were represented. The instruments were administered in 11 languages, most commonly English (*n* = 10) followed by Chinese (*n* = 5) and Spanish (*n* = 5). Instruments were translated from the original language in 20 (63%) studies. Only one study was conducted with Indigenous peoples [21].

Psychometric properties of the instruments most frequently assessed were internal consistency, structural validity and hypothesis testing (see Table 2). One study assessed measurement error and no studies assessed responsiveness. Assessment of methodological quality is presented in Table 3. The results of each study on the assessed measurement properties (following COSMIN guidelines of good measurement properties; [15]) are presented for each individual study and pooled results where multiple studies assessed a single instrument in Table 4. In what follows, we provide a summary of the psychometric properties of the instruments across the seven constructs.

### Psychological capacity constructs

#### Resilience

Resilience was the most measured construct, with 11 instruments measured across 19 studies. As with the other constructs of psychological capacity identified in the review, there is a lack of definitional consistency relating to resilience. However, it can be broadly understood as ‘a dynamic process that results in positive adaptation when faced with adversity’ [35](p. 215). Three instruments were measured in multiple studies—the Connor–Davidson Resilience Scale (CDRS), the Wagnild–Young Resilience Scale and the Brief Resilient Coping Scale. The CDRS was assessed in five studies involving 5,215 participants. Two studies administered the CDRS in the original language (English) and three administered translated versions. Overall, the CDRS demonstrated sufficient reliability, criterion validity and cross-cultural validity/measurement invariance but insufficient validity across the five studies focusing on this instrument. A short form of the scale (10 items) showed sufficient reliability and validity in one of the studies [21].

The Wagnild–Young Resilience Scale was assessed in four studies involving 4,536 participants. Two studies administered it in the original language (English) and one a translated version. Overall, the scale demonstrated insufficient reliability and structural validity and sufficient hypothesis testing and cross-cultural validity/measurement invariance. A short form of the scale (5 items) showed sufficient reliability and validity in one of the studies [33].

The Brief Resilient Coping Scale was assessed in three studies involving 1,360 participants. All administered translated versions (Spanish). Overall, the scale demonstrated sufficient internal reliability, structural validity and cross-cultural validity/measurement invariance, with hypothesis testing assessed as indeterminate. Of the remaining eight instruments, the Resilience Scale for Oldest-Old Age, The University of Washington Resilience Scale, the Multidimensional Individual and Interpersonal Resilience Measure, and the Resilience Scale for Older Adults all showed sufficient reliability and validity in older people.

**Table 1:** Characteristics of the included studies

Author (date)/country	Name of the measure(s)	Language/original or translation	Study design	Study population	Setting	Measurement properties		
						Validity	Reliability	Other
Resilience								
Akatsuka & Tadaka (2021) [22] Japan	Resilience Scale for Oldest-old age (RSO)	Japanese/original	Cross-sectional	*n* = 1,283, mean age = 84.7, M = 55.4%, F = 44.6%, 29.0% were certificated for long-term care need, mean number of diseases = 1.8	Community	√	√	
Amtmann *et al*. (2020) [18] USA	Resilience Item Bank and Short Form	English/original	Cross-sectional	People with chronic medical conditions: *n* = 1,441, mean age = 63, M = 35%, F = 65%; general population: *n* = 300; mean age = 49, M = 49%, F = 51%	Community	√	√	
Cohen *et al.* (2017) [23] USA	Wagnild–Young Resilience Scale; Windle–Markland–Woods Psychological Resilience Scale; Connor–Davidson Resilience Scale	English/original	Cross-sectional	*n* = 200 older adults, mean age = 80.9 and *n* = 192 youth, mean age = 13.2	Community	√	√	
da Silva-Sauer *et al*. (2021) [24] Brazil	Brief Resilience Scale	Brazilian-Portuguese/ translation	Cross-sectional	*n* = 1,251, mean age = 68.02 (SD = 6.52), M = 45.8%, F = 54.2%	Community	√	√	
Goins *et al*. (2012) [21] USA	Connor–Davidson Resilience Scale	English (older American Indians)/original	Cross-sectional	*n* = 160, mean age = 67.9 (SD = 9.9), M = 31.2%, F = 68.8%, 35.6% diagnosed with diabetes, 31.3% diagnosed with cardiovascular disease, 43.8% had at least one activity of daily living limitation	Community	√	√	
Hawkley *et al*. (2021) [17] USA	4-item resilience scale	English/original	Cross-sectional	*n* = 4,604, aged between 50–59 and 80–95	Community	√	√	√
Li & Ow (2022) Taiwan [25]	Resilience Scale for Older Adults (RSOA)	Chinese/original	Cross-sectional	*n* = 368, 38.3% aged between 71 and 80, 31.8% aged between 81 and 91, M = 32.9%, F = 67.1%	Aged care facility	√	√	
Martin *et al*. (2015) [26] USA	Multidimensional Individual and Interpersonal Resilience Measure (MIIRM)	English/original	Cross-sectional	*n* = 1,006, mean age = 77.35 (SD = 12.2), M = 51.4%, F = 48.6%	Community	√	√	
Meng *et al*. (2019) [27] China	Connor–Davidson Resilience Scale	Chinese/translation	Cross-sectional	*n* = 1,238, mean age = 72.47 (SD = 9.09), M = 42%, F = 58%	Community	√	√	
Moret-Tatay *et al*. (2015) [19] Spain	Brief Resilient Coping Scale	Spanish/translation	Cross-sectional	*n* = 991, mean age = 62.7 (SD = 5.89), M = 75.9%, F = 24.2%	Community	√	√	
Resnick & Inguito (2011) [28] USA	The Resilience Scale (Wagnild–Young)	English/original	Cross-sectional	*n* = 163 residents in a continuing care retirement community setting (mean age = 86.3, SD = 5.8), M = 26%, F = 74%; *n* = 101 older women post-hip fracture (mean age = 80, SD = 7.6), M = 0%, F = 100%	Community	√	√	
Tomas *et al*. (2012) [29] Spain	Brief Resilient Coping Scale	Spanish/translation	Cross-sectional	*n* = 133, mean age = 71.7 (SD = 6.93), M = 30% F = 70%	Community	√	√	
Tomas *et al*. (2021) [30] Spain	Brief Resilient Coping Scale	Spanish/translation	Cross-sectional	Peru sample = 236, mean age = 72.8 (SD = 6.9), M = 21.6% F = 78.4%; Spain sample = 133, mean age = 71 (SD = 7), M = 30.1%, F = 69.9%	Community	√	√	
Tourunen *et al.* (2021) [31] Finland	Connor–Davidson Resilience Scale	Finnish/translation	Cross-sectional	*n* = 1,018, age 75 (45%), 80 (33%) and 85 (22%), M = 43%, F = 57%	Community	√	√	
Velickovic *et al*. (2020) [32] Sweden	Connor–Davidson Resilience Scale	Swedish/translation	Cross-sectional	*n* = 2,599, age 45–54 = 330, 55–64 = 737, 65–74 = 1,283, 75–84 = 249, M = 1,316, F = 1,283	Community	√	√	√
von Eisenhart Rothe *et al*. (2013) [33] Germany	Resilience Scale (Wagnild & Young)	German/translation	Cross-sectional	*n* = 3,712, median age = 72 (SD = 5.8), M = 48%, F = 52%	Community	√	√	
Whitehall *et al*. (2021) [34] Scotland	Making it Clear (MiC)	English/original	Cross-sectional	*n* = 416, mean age 85.33 (SD = 6.54), M = 134, F = 282	Hospital	√	√	
Wilson *et al.* (2022) [35] Canada	Resilience Scale for Older Adults	English/original	Cross-sectional	Study 1: *n* = 345, mean age = 65.32 (SD = 4.54), M = 112, F = 231; Study 2: *n* = 216, mean age = 71.55 (SD = 7.78), M = 73, F = 142; Study 3: *n* = 365, mean age = 64.01 (SD = 5.31), M = 111, F = 253	Community	√	√	
Yang *et al*. (2015) [36] China	Resilience Scale (Wagnild–Young)	Chinese/translation	Cross-sectional	*n* = 461, age 60–88, M = 270; F = 191	Community	√	√	
Sense of coherence								
McGee *et al.* (2018) [37] Switzerland	Sense of Coherence scale	German/translation	Cross-sectional	*n* = 268, mean age 66.94 (SD = 8.96), M = 77, F = 191	Community	√	√	√
Naaldenberg *et al*. (2011) [38] The Netherlands	Orientation to Life Questionnaire (OLQ-13)	Dutch/translation	Cross-sectional	*n* = 1,361, mean age = 75 (SD = 6.8), M = 43%, F = 57%	Community	√	√	
Söderhamn *et al*. (2015) [39] Norway	Sense of Coherence Scale	Norwegian/translation	Cross-sectional	*n* = 2069, mean age = 74.5 (SD = 6.9), M = 49.8%, F = 50.2%	Community	√	√	
von Humboldt & Leal (2015) [40] Portugal	Orientation to Life Questionnaire (OtLQ)	Portuguese/translation	Cross-sectional	*n* = 1,291, mean age = 83.9 (SD = 6.68), M = 540, F = 751	Community	√	√	
Hope								
Chan *et al.* (2012) [41] Hong Kong	Herth Hope Index	Chinese/translation	Cross-sectional	*n* = 120, aged 60–80, M = 56.7%, F = 42.3%; *n* = 24 participated in retest; All patients with heart failure	Hospital	√	√	
DiGasbarro *et al*. (2020) [16] USA	Adult Hope Scale	English/original	Cross-sectional	*n* = 64, mean age = 74.45 (SD = 10.49), M = 21, F = 43; 32 had impaired cognitive functioning	Aged care facility	√	√	
Haugan *et al*. (2013) [20] Norway	Herth Hope Index	Norwegian/translation	Cross-sectional	*n* = 202, mean age = 85.87, M = 27.7%, F = 72.3%; Mild–moderate depression (30%); anxiety (12%)	Aged care facility	√	√	
Yaghoobzadeh *et al.* (2019) [42] Iran	Herth Hope Index	Persian/translation	Cross-sectional	*n* = 500, mean age = 66.2 (SD = 5.76), M = 214, F = 290	Primary care	√	√	
Mindfulness								
Brady *et al*. (2019) [43] Australia	Five Facet Mindfulness Questionnaire	English/original	Cross-sectional	*n* = 210, mean age = 65.51, M = 55%, F = 45%; Five participants reported experiencing at least one stroke. Nine participants reported the presence of a current mood disorder	Community	√	√	
Caycho-Rodríguez *et al*. (2021) [44] Peru	Mindful Attention Awareness Scale (MAAS-5)	Spanish/translation	Cross-sectional	*n* = 323, mean age F = 68.58 (SD = 7.23), mean age M = 68.91 (SD = 7.12), M = 162, F = 160	Community	√	√	
Optimism								
Huang *et al*. (2020) [45] Canada	Chinese version of the Revised Life Orientation Test (CLOT-R)	Chinese/translation	Cross-sectional	*n* = 342, mean age = 71.99 (SD = 5.62), M = 41.5%, F = 58.50%	Community	√	√	
Attachment to Life								
Araújo *et al.* (2015) [46] Portugal	Positive Valuation of Life Scale (Positive VOL)	Portuguese/translation	Cross-sectional	*n* = 207, mean age 77.2 (SD = 7.5), M = 78, F = 129	Community	√	√	
Emotional Regulation								
Carvajal *et al*. (2022) [47] Spain	Cognitive Emotion Regulation Questionnaire (CERQ)	Spanish/translation	Cohort	*n* = 305, mean age = 70.02 (SD = 4.67), M = 65.25%, F = 34.75%. *n* = 150 took part in the second wave of the study after a follow-up period of 6 months	Community	√	√	√

**Table 2:** Psychometric properties assessed for each measure

Name of measure	Psychometric properties assessed in the included studies								
	Internal consistency	Reliability	Measurement error	Structural validity	Hypothesis testing	Cross-cultural validity/ measurement invariance	Criterion validity	Responsiveness	Other
Resilience									
Connor–Davidson Resilience Scale	√	√		√	√	√	√		√
Brief Resilient Coping Scale	√			√	√	√			
Wagnild–Young Resilience Scale	√	√		√	√	√			
University of Washington Resilience Scale (UWRS)	√			√	√		√		
Windle–Markland–Woods Psychological Resilience	√			√					
Resilience Scale for Oldest-old age (RSO)	√			√	√				
Brief Resilience Scale	√			√	√	√			
4-item resilience scale	√				√				√
Resilience Scale for Older Adults (RSOA)	√	√	√	√	√		√		
Multidimensional Individual and Interpersonal Resilience Measure (MIIRM)	√			√		√	√		
Making it Clear (MiC)	√			√	√				
Resilience Scale for Older Adults	√			√	√	√			
Sense of coherence									
Sense of Coherence Scale	√			√	√	√			√
Orientation to Life Questionnaire	√	√		√	√				
Hope									
Herth Hope Index	√	√		√	√				
Adult Hope Scale	√				√				
Mindfulness									
Five Facet Mindfulness Questionnaire	√			√	√				
Mindful Attention Awareness Scale (MAAS-5)	√			√	√	√			
Optimism									
Chinese version of the Revised Life Orientation Test (CLOT-R)	√			√	√				
Attachment to life									
Positive Valuation of Life Scale (Positive VOL)	√			√	√				
Emotional regulation									
Cognitive Emotion Regulation Questionnaire (CERQ)	√	√		√	√				√

**Table 3:** Risk-of-bias ratings

Name of measure	Psychometric properties of the instruments included and rated by the COSMIN checklist									
Author	Patient reported outcome measures development	Content validity	Internal consistency	Reliability	Measurement error	Structural validity	Hypothesis testing	Cross-cultural validity/ measurement Invariance	Criterion validity	Responsiveness
Resilience (*n* = 19 articles)										
Connor–Davidson Resilience Scale										
Cohen *et al*. (2017)^*^ [23]			Very good			Very good				
Goins *et al*. (2012) [21]	Very good		Very good			Very good	Very good		Very good	
Meng *et al*. (2019) [27]			Very good	Adequate		Very good		Very good		
Tourunen *et al*. (2021) [31]			Very good	Inadequate		Adequate	Doubtful			
Velickovic *et al.* (2020) [32]			Very good			Very good	Doubtful			
Brief Resilient Coping Scale										
Moret-Tatay *et al*. (2015) [19]			Very good			Very good	Very good			
Tomas *et al*. (2012) [29]			Very good			Very good	Adequate			
Tomas *et al*. (2021) [30]			Very good			Very good		Adequate		
Wagnild–Young Resilience Scale										
Cohen *et al*. (2017)^*^ [23]			Very good			Very good				
Resnick & Inguito (2011) [28]			Very good	Inadequate		Adequate	Very good			
von Eisenhart Rothe *et al*. (2013) [33]			Very good			Very good	Very good	Very good	Very good	
Yang *et al*. (2015) [36]	Adequate	Doubtful	Very good	Adequate		Adequate				
Resilience Scale for Oldest-Old Age (RSO)										
Akatsuka & Tadaka (2021) [22]			Very good			Very good	Very good		Very good	
University of Washington Resilience Scale (UWRS)										
Amtmann *et al*. (2020) [18]	Very good	Adequate	Very good			Very good	Very good			
Windle–Markland–Woods Psychological Resilience										
Cohen *et al*. (2017)^*^ [23]			Very good			Very good				
Brief Resilience Scale										
da Silva-Sauer *et al*. (2021) [24]	Inadequate		Very good			Very good	Very good	Very good		
4-item Resilience scale										
Hawkley *et al*. (2021) [17]			Very good			Very good				
Resilience Scale for Older Adults (RSOA)										
Li & Ow (2022) [25]	Very good	Adequate	Very good	Adequate	Adequate	Very good	Doubtful		Very good	
Multidimensional Individual and Interpersonal Resilience Measure (MIIRM)										
Martin *et al*. (2015) [26]	Very good		Very good			Very good		Very good	Very good	
Making it Clear (MiC)										
Whitehall *et al*. (2021) [34]			Very good			Adequate	Adequate			
Resilience Scale for Older Adults										
Wilson *et al*. (2022) [35]			Very good			Very good	Doubtful	Very good		
Sense of Coherence (*n* = 4 articles)										
Sense of Coherence Scale										
McGee *et al*. (2018) [37]			Very good			Very good	Adequate	Adequate		
von Humboldt & Leal (2015) [40]			Very good			Very good	Very good			
Orientation to Life Questionnaire										
Naaldenberg *et al*. (2011) [38]		Doubtful	Very good	Adequate		Doubtful				
Söderhamn *et al*. (2015) [39]			Very good			Very good	Very good			
Hope (*n* = 4 articles)										
Herth Hope Index										
Chan *et al*. (2012) [41]			Very good	Very good		Very good	Very good			
Haugan *et al*. (2013) [20]			Very good			Very good	Very good			
Yaghoobzadeh *et al*. (2019) [42]			Very good			Very good				
Adult Hope Scale										
DiGasbarro *et al*. (2020) [16]			Adequate				Very good			
Mindfulness (*n* = 2 articles)										
Five Facet Mindfulness Questionnaire										
Brady *et al*. (2019) [43]			Doubtful			Inadequate	Very good			
Mindful Attention Awareness Scale (MAAS-5)										
Caycho-Rodríguez *et al*. (2021) [44]			Inadequate			Very good	Very good	Very good		
Optimism										
Chinese version of the Revised Life Orientation Test (CLOT-R)										
Huang *et al*. (2020) [45]			Very good			Very good	Adequate			
Attachment to Life										
Positive Valuation of Life Scale (Positive VOL)										
Araújo *et al.* (2015) [46]			Very good			Very good	Very good			
Emotional Regulation										
Cognitive Emotion Regulation Questionnaire (CERQ)										
Carvajal *et al*. (2022) [47]			Very good	Very good		Very good	Very good			

**Table 4:** Summary of validity and reliability of the instruments

Name of measure	Summary of validity and reliability + = sufficient, − = insufficient, ? = indeterminate
Author	
Resilience (*n* = 19 articles)	
Connor–Davidson Resilience Scale Internal reliability + Reliability ? Structural validity − Hypothesis testing − Criterion validity + Cross-cultural validity/measurement invariance +	
Cohen *et al*. (2017)^a^ [23]	Internal reliability + Structural validity −
Goins *et al*. (2012) [21]	Internal reliability + Structural validity − Criterion validity + Cross-cultural validity/measurement invariance + 10-item version: Internal reliability + Structural validity + Criterion validity + Cross-cultural validity/measurement invariance +
Meng *et al*. (2019) [27]	Internal reliability + Reliability ? Structural validity + Cross-cultural validity/measurement invariance +
Tourunen *et al*. (2021) [31]	Internal reliability + Reliability − Structural validity − Hypothesis testing +
Velickovic *et al*. (2020) [32]	Internal reliability + Structural validity + Hypothesis testing ?
Brief Resilient Coping Scale Internal reliability + Structural validity + Hypothesis testing? Cross-cultural validity/measurement invariance +	
Moret-Tatay *et al*. (2015) [19]	Internal reliability + Structural validity + Hypothesis testing ?
Tomas *et al*. (2012) [29]	Internal reliability + Structural validity + Hypothesis testing?
Tomas *et al*. (2021) [30]	Internal reliability + Structural validity + Cross-cultural validity/measurement invariance +
Wagnild–Young Resilience Scale Internal reliability – Reliability ? Structural validity − Hypothesis testing + Cross-cultural validity/measurement invariance +	
Cohen *et al.* (2017)^a^ [23]	Internal reliability + Structural validity −
Resnick & Inguito (2011) [28]	Internal reliability + Reliability? Structural validity − Hypothesis testing +
von Eisenhart Rothe *et al*. (2013) [33]	RS-11: Internal reliability + Structural validity − Hypothesis testing + Cross-cultural validity/measurement invariance + RS-5 (short form): Internal reliability + Structural validity + Hypothesis testing + Cross-cultural validity/measurement invariance + Criterion validity +
Yang *et al*. (2015) [36]	Internal reliability + Reliability ? Structural validity −
Resilience Scale for Oldest-Old Age (RSO)	
Akatsuka & Tadaka (2021) [22]	Internal reliability + Structural validity + Hypothesis testing +
University of Washington Resilience Scale (UWRS)	
Amtmann *et al*. (2020) [18]	Internal reliability + Structural validity + Hypothesis testing + Criterion validity +
Windle–Markland–Woods Psychological Resilience Scale	
Cohen *et al.* (2017)^a^ [23]	Internal reliability − Structural validity −
Brief Resilience Scale	
da Silva-Sauer *et al*. (2021) [24]	Internal reliability + Structural validity + Hypothesis testing? Cross-cultural validity/measurement invariance −
4-Item resilience scale	
Hawkley *et al*. (2021) [17]	Internal reliability + Hypothesis testing?
Resilience Scale for Older Adults (RSOA)	
Li & Ow (2022) [25]	Internal consistency + Reliability ? Measurement error ? Structural validity + Hypothesis testing ? Criterion validity +
Multidimensional Individual and Interpersonal Resilience Measure (MIIRM)	
Martin *et al*. (2015) [26]	Internal consistency + Structural validity + Cross-cultural validity/measurement invariance + Criterion validity +
Making it Clear (MiC)	
Whitehall *et al*. (2021) [34]	Internal consistency + Structural validity – Hypothesis testing?
Resilience Scale for Older Adults	
Wilson *et al*. (2022) [35]	Internal consistency + Structural validity + Hypothesis testing + Cross-cultural validity/measurement invariance +
Sense of Coherence (*n* = 4 articles)	
Sense of Coherence Scale Internal consistency + Structural validity + Hypothesis testing ? Cross-cultural validity/measurement invariance +	
McGee *et al*. (2018) [37]	Internal consistency + Structural validity + Hypothesis testing ? Cross-cultural validity/measurement invariance +
von Humboldt & Leal (2015) [40]	Internal consistency + Structural validity + Hypothesis testing +
Orientation to Life Questionnaire Internal consistency + Reliability ? Structural validity + Hypothesis testing +	
Naaldenberg *et al*. (2011) [38]	Internal consistency + Reliability ? Structural validity +
Söderhamn *et al*. (2015) [39]	Internal consistency + Structural validity + Hypothesis testing +
Hope (*n* = 4 articles)	
Herth Hope Index Internal consistency + Reliability + Structural validity + Hypothesis testing +	
Chan *et al*. (2012) [41]	Internal consistency + Reliability + Structural validity + Hypothesis testing +
Haugan *et al*. (2013) [20]	Internal consistency + Structural validity + Hypothesis testing +
Yaghoobzadeh *et al*. (2019) [42]	Internal consistency + Structural validity +
Adult Hope Scale	
DiGasbarro *et al*. (2020) [16]	Internal consistency + Hypothesis testing +
Mindfulness (*n* = 2 articles)	
Five Facet Mindfulness Questionnaire	
Brady *et al*. (2019) [43]	Internal consistency + Structural validity + Hypothesis testing +
Mindful Attention Awareness Scale (MAAS-5)	
Caycho-Rodríguez *et al*. (2021) [44]	Internal consistency + Structural validity + Hypothesis testing + Cross-cultural validity/measurement invariance +
Optimism	
Chinese version of the Revised Life Orientation Test (CLOT-R)	
Huang *et al*. (2020) [45]	Internal consistency + Structural validity + Hypothesis testing ?
Attachment to Life	
Positive Valuation of Life Scale (Positive VOL)	
Araújo *et al*. (2015) [46]	Internal consistency + Structural validity − Hypothesis testing +
Emotional Regulation	
Cognitive Emotion Regulation Questionnaire (CERQ)	
Carvajal *et al*. (2022) [47]	Internal consistency + Reliability + Structural validity + Hypothesis testing +

^a^Assessed three instruments

#### Sense of coherence

Sense of coherence, defined as the ‘the ability to identify and utilise internal and external resources to facilitate successful coping with stressors and maintain and develop health’ [37] (p. 1438), was measured by two instruments. The Sense of Coherence Scale was assessed in translated versions in two studies involving 2,337 participants. The scale demonstrated sufficient internal consistency, structural validity and cross-cultural validity/measurement invariance with indeterminate hypothesis testing. The Orientation to Life Questionnaire was also assessed in translated versions in two studies involving 2,652 participants. The scale demonstrated sufficient internal consistency, structural validity and hypothesis testing with indeterminate reliability.

#### Hope

Hope, which can be defined as ‘a multidimensional dynamic life force characterized by a confident, yet uncertain expectation of achieving something good’ [20] (p. 379), was measured by two instruments. The Herth Hope Index was assessed in translated versions in three studies involving 822 participants. The scale demonstrated sufficient internal consistency, reliability, structural validity and hypothesis testing. The Adult Hope Scale was assessed in original English language in one study with 64 participants, demonstrating sufficient internal consistency and hypothesis testing.

#### Mindfulness

Mindfulness, defined as a ‘non-judgmental present-moment awareness and attention’ [43] (p. 549), was measured by two instruments. The Five Facet Mindfulness Questionnaire was assessed in original English language in one study with 210 participants and demonstrated sufficient internal consistency, structural validity and hypothesis testing. The Mindful Attention Awareness Scale was assessed in a translated version with 323 participants and demonstrated sufficient internal consistency, structural validity, hypothesis testing and cross-cultural validity/measurement invariance.

#### Optimism

Optimism can be defined as ‘having a positive outcome expectancy for the future’ [20] (p. 2618). One instrument measuring optimism was identified. The Chinese version of the Revised Life Orientation Test was assessed in a translated version with 342 participants and demonstrated sufficient internal consistency and structural validity but indeterminate hypothesis testing.

#### Attachment to life

Attachment to life is ‘the subjectively experienced worth of a person’s life’ [46] (p. 2385). One instrument measuring attachment to life was identified. The Positive Valuation of Life Scale was assessed in a translated version with 207 participants and demonstrated sufficient internal consistency and hypothesis testing but insufficient structural validity.

#### Emotional regulation

Emotional regulation is linked to coping and includes ‘conscious efforts to regulate one’s emotions in the face of stressful situations’ [47] (p. 413). One instrument measuring emotional regulation was identified. The Cognitive Emotion Regulation Questionnaire was assessed in a translated version with 305 participants and demonstrated sufficient internal consistency, reliability, structural validity and hypothesis testing.

## Discussion

Psychological capacity is an important domain of the intrinsic capacity component of healthy ageing identified by WHO [2]. This systematic review builds on the discussion of aspects of psychological capacity in older people and how this could be measured following the WHO baseline report [2]. No single measure encompassing the broad concept of intrinsic psychological capacity was identified in the review. The review identified 20 instruments measuring 7 constructs of psychological capacity.

The most common definitions of psychological capacity come from the positive psychology literature and include hope, optimism, self-efficacy and resilience [7]. We identified instruments measuring resilience, hope and optimism in older people; however, measures of self-efficacy were excluded as they measured self-efficacy in relation to various diseases/conditions and no studies of the psychometric properties of measures of general self-efficacy for older people were identified. We also identified measures of additional constructs that are consistent with the attributes of psychological capacity, including sense of coherence, mindfulness, attachment to life and emotional regulation.

Resilience was the most widely measured construct in older people with a range of psychometric properties evaluated. Other constructs were limited to only one or two studies with further work needed to determine their validity and reliability. There was also limited research conducted in lower middle-income countries and no psychometric research on psychological capacity was identified from low-income countries. This might be due to the exclusion of non-English language studies, with 16 studies excluded for this reason. These were published in Spanish (*n* = 9), Portuguese (*n* = 2), Persian (*n* = 1), Japanese (*n* = 1) and Finnish (*n* = 1). Of these, eight measured constructs of psychological capacity consistent with our inclusion criteria.

The definitions of the included constructs show an interconnection across constructs and a relationship between intrinsic capacity, life experience and environmental factors. There is an apparent relationship between the identified constructs and resilience, suggesting that resilience might be an overarching construct of psychological capacity in older people. However, there is a need for further work to better define psychological capacity and develop a single measure for the purposes of WHO reporting.

## Limitations

A major limitation of this study was lack of a clear definition of psychological capacity, which is an area for future research. For this review, we understood aspects of psychological capacity as positive states of mind that enable individuals to enhance their physical, mental and social functioning. However, we acknowledge there is potential variability and overlap in the dimensionality of these constructs. For example, resilience is a heterogenous construct and may overlap with self-efficacy and control. In addition, due to limited resources a further limitation was the exclusion of non-English language studies. We also acknowledge that the limited evidence from low–middle-income countries may restrict our understanding of psychometric properties of the aspects of psychological capacities in these countries. Future psychometric studies on psychological capacity should equitably include low- and middle-income countries and thereby ensure cross-cultural validity and applicability.

## Conclusion

This systematic review has identified significant knowledge gaps in the measurement of psychological capacity in older people. The lack of definitional clarity of the concept of psychological capacity limits our ability to form a clear conclusion on the most appropriate measure. While the concept of resilience stands out as the most widely understood and researched concept in the psychometric literature, further work is needed to clarify the concept of psychological capacity and how this could be measured in older people. This is currently underway through expert consultation to develop a more definitive definition of the concept of psychological capacity, underpinned by the findings of this systematic review.

## Supplementary Material

aa-23-0342-File001_afad100

## Data Availability

The data that support the findings of this study are available in the Supplementary materials Appendix 1.
