# Supplementary material for: A systematic review of the measurement properties of aspects of psychological capacity in older adults
Source: Age Ageing. 2023 Oct 30;52(Suppl 4):iv67–81. doi: 10.1093/ageing/afad100 (PMC10615039; doi:10.1093/ageing/afad100)
Supplement: aa-23-0342-File001_afad100 [file aa-23-0342-file001_afad100.docx]

World Health Organization: *Measurement of Healthy Ageing.*

**A systematic review of the measurement properties of aspects of psychological capacity in older adults.**

SUPPLEMENTARY DATA

**Appendix 1.** Search Terms

| 1 exp aging/ or exp Older Adulthood/ or Middle Aged/ |
| --- |
| 2 (advanced years or ageing or aging or elder* or frail or geriatric* or gerontology* or middle aged or midlife or old age or oldest old or pensioner* or post?menopausal or postmenopausal or “senior citizen”*”).ti, ab, id. |
| 3 (older adj3 (adult* or female* or male* or men or people or person or women)).ti, ab, id. |
| 4 1 or 2 or 3 |
| 5 exp psychometrics/ or measurement/ or exp Test Reliability/ or exp Test Validity/ or exp Test Construction/ or exp Rating Scales/ |
| 6 (psychometric* or validation or “construct validity” or rating scale* or (test adj2 (valid* or construction))).ti, ab, id. |
| 7 5 0 6 |
| 8 positive psychology/ or psychological capital/ or hope/ or optimism/ or “resilience (psychological)”/ or self-efficacy/ or exp “Resilience (Psychological)”/ or exp “Quality of Life”/ or exp Healthy Aging/ or exp “Aging (Attitudes Toward)”/ or Adaptation, Psychological/ or exp Spirituality/ or exp Humor/ or exp Coping Behavior/ or exp Creativity/ or exp Participation/ or exp Social Interaction/ or exp Community Involvement/ or exp Social Networks/ or exp Mindfulness/ or sympathy/ or exp Love/ or wisdom/ |
| 9 (positive psychology or psychological capital or Self-efficacy or Optimism or Hope or Life satisfaction or Happiness or Self-control of Intelligence or Independence or Adaptive coping or Self-rates successful ageing or resilience of religiousness or spirituality or life evaluation or resourcefulness or sense of coherence or autonomy or hope or dignity or love or compassion or sense of agency or gratitude or pleasure or self-realization or cope or wisdom or growth or transcendence or social participation or social inclusion or self-concept or humour or creativity or flow or benefit finding or opportunity or community integration or mindfulness or acceptance or social adjustment or locus of control or psychological well-being).ti, ab, id. |
| 10 8 or 9 |
| 11 4 and 7 and 10 |
